# Supplementary material for: Scope of Use and Effectiveness of Dietary Interventions for Improving Health-Related Outcomes in Veterans: A Systematic Review
Source: Nutrients. 2022 May 17;14(10):2094. doi: 10.3390/nu14102094 (PMC9147269; doi:10.3390/nu14102094)
Supplement: Supplementary file 1 [file nutrients-14-02094-s001.zip › nutrients-1686237-supplementary.pdf]

Supplementary Materials

Scope of use and effectiveness of dietary interventions for improving health-related outcomes in veterans: a systematic review

Supplementary Material 1

Table S1. PRISMA 2020 Checklist

Supplementary Material 2

Final executed search strategies for relevant databases

Supplementary Material 3

Table S2. Main Characteristics of Studies that Included MOVE! Weight loss program as the intervention group or the control/comparison group, ordered and grouped by comorbid health condition (in addition to meeting eligibility criteria for MOVE! program).

Table S3. Health-related Outcome Measures in studies that included MOVE! Weight loss program as the intervention group or the control/comparison group, ordered and grouped by comorbid health condition (in addition to meeting eligibility criteria for MOVE! program).

PRISMA 2020 Checklist

Section and Topic	Item #	Checklist item	Location where item is reported
**TITLE**			
Title	1	Identify the report as a systematic review.	P.1
**ABSTRACT**			
Abstract	2	See the PRISMA 2020 for Abstracts checklist.	P.1
**INTRODUCTION**			
Rationale	3	Describe the rationale for the review in the context of existing knowledge.	P.2
Objectives	4	Provide an explicit statement of the objective(s) or question(s) the review addresses.	P.2
**METHODS**			
Eligibility criteria	5	Specify the inclusion and exclusion criteria for the review and how studies were grouped for the syntheses.	P.3
Information sources	6	Specify all databases, registers, websites, organisations, reference lists and other sources searched or consulted to identify studies. Specify the date when each source was last searched or consulted.	P.2
Search strategy	7	Present the full search strategies for all databases, registers and websites, including any filters and limits used.	Supplementary Material 2
Selection process	8	Specify the methods used to decide whether a study met the inclusion criteria of the review, including how many reviewers screened each record and each report retrieved, whether they worked independently, and if applicable, details of automation tools used in the process.	P.4
Data collection process	9	Specify the methods used to collect data from reports, including how many reviewers collected data from each report, whether they worked independently, any processes for obtaining or confirming data from study investigators, and if applicable, details of automation tools used in the process.	P.4
Data items	10a	List and define all outcomes for which data were sought. Specify whether all results that were compatible with each outcome domain in each study were sought (e.g. for all measures, time points, analyses), and if not, the methods used to decide which results to collect.	P.3
	10b	List and define all other variables for which data were sought (e.g. participant and intervention characteristics, funding sources). Describe any assumptions made about any missing or unclear information.	
Study risk of bias assessment	11	Specify the methods used to assess risk of bias in the included studies, including details of the tool(s) used, how many reviewers assessed each study and whether they worked independently, and if applicable, details of automation tools used in the process.	P.4
Effect measures	12	Specify for each outcome the effect measure(s) (e.g. risk ratio, mean difference) used in the synthesis or presentation of results.	N/A
Synthesis methods	13a	Describe the processes used to decide which studies were eligible for each synthesis (e.g. tabulating the study intervention characteristics and comparing against the planned groups for each synthesis (item #5)).	N/A
	13b	Describe any methods required to prepare the data for presentation or synthesis, such as handling of missing summary statistics, or data conversions.	N/A
	13c	Describe any methods used to tabulate or visually display results of individual studies and syntheses.	N/A
	13d	Describe any methods used to synthesize results and provide a rationale for the choice(s). If meta-analysis was performed, describe the model(s), method(s) to identify the presence and extent of statistical heterogeneity, and software package(s) used.	N/A
	13e	Describe any methods used to explore possible causes of heterogeneity among study results (e.g. subgroup analysis, meta-regression).	N/A
	13f	Describe any sensitivity analyses conducted to assess robustness of the synthesized results.	N/A
Reporting bias assessment	14	Describe any methods used to assess risk of bias due to missing results in a synthesis (arising from reporting biases).	N/A
Certainty assessment	15	Describe any methods used to assess certainty (or confidence) in the body of evidence for an outcome.	N/A

PRISMA 2020 Checklist

Section and Topic	Item #	Checklist item	Location where item is reported
**RESULTS**			
Study selection	16a	Describe the results of the search and selection process, from the number of records identified in the search to the number of studies included in the review, ideally using a flow diagram.	P.5
	16b	Cite studies that might appear to meet the inclusion criteria, but which were excluded, and explain why they were excluded.	N/A
Study characteristics	17	Cite each included study and present its characteristics.	Line 201
Risk of bias in studies	18	Present assessments of risk of bias for each included study.	Line 186
Results of individual studies	19	For all outcomes, present, for each study: (a) summary statistics for each group (where appropriate) and (b) an effect estimate and its precision (e.g. confidence/credible interval), ideally using structured tables or plots.	N/A
Results of syntheses	20a	For each synthesis, briefly summarise the characteristics and risk of bias among contributing studies.	N/A
	20b	Present results of all statistical syntheses conducted. If meta-analysis was done, present for each the summary estimate and its precision (e.g. confidence/credible interval) and measures of statistical heterogeneity. If comparing groups, describe the direction of the effect.	N/A
	20c	Present results of all investigations of possible causes of heterogeneity among study results.	N/A
	20d	Present results of all sensitivity analyses conducted to assess the robustness of the synthesized results.	N/A
Reporting biases	21	Present assessments of risk of bias due to missing results (arising from reporting biases) for each synthesis assessed.	N/A
Certainty of evidence	22	Present assessments of certainty (or confidence) in the body of evidence for each outcome assessed.	N/A
**DISCUSSION**			
Discussion	23a	Provide a general interpretation of the results in the context of other evidence.	Line 416+
	23b	Discuss any limitations of the evidence included in the review.	Line 559+
	23c	Discuss any limitations of the review processes used.	a/a
	23d	Discuss implications of the results for practice, policy, and future research.	Line 589+
**OTHER INFORMATION**			
Registration and protocol	24a	Provide registration information for the review, including register name and registration number, or state that the review was not registered.	P.2
	24b	Indicate where the review protocol can be accessed, or state that a protocol was not prepared.	P.2
	24c	Describe and explain any amendments to information provided at registration or in the protocol.	N/A
Support	25	Describe sources of financial or non-financial support for the review, and the role of the funders or sponsors in the review.	Line 623
Competing interests	26	Declare any competing interests of review authors.	Line 629
Availability of data, code and other materials	27	Report which of the following are publicly available and where they can be found: template data collection forms; data extracted from included studies; data used for all analyses; analytic code; any other materials used in the review.	

<u>Supplementary Material 2:</u>

Final executed search strategies for relevant databases

MEDLINE via PubMed

("veterans"[MeSH Terms] OR "veterans health"[MeSH Terms] OR "veterans health services"[MeSH Terms] OR "united states department of veterans affairs"[MeSH Terms] OR "veteran*"[Title/Abstract] OR "ex-service"[Title/Abstract] OR "ex-forces"[Title/Abstract] OR "ex- military"[Title/Abstract] OR "military transitions"[Title/Abstract] OR "military to civilian"[Title/Abstract]) AND ("diet*"[Title/Abstract] OR "dietetic*"[Title/Abstract] OR "dietitian"[Title/Abstract] OR "dietician"[Title/Abstract] OR "eating pattern"[Title/Abstract] OR "eating patterns"[Title/Abstract] OR "eating habit"[Title/Abstract] OR "eating habits"[Title/Abstract] OR "dietary pattern"[Title/Abstract] OR "dietary patterns"[Title/Abstract] OR "dietary quality"[Title/Abstract] OR "nutrition*"[Title/Abstract] OR "lifestyle"[Title/Abstract] OR "weight loss"[Title/Abstract] OR "weight management"[Title/Abstract] OR "healthy eating"[Title/Abstract] OR "diet"[MeSH Terms])

CINAHL

((MH "veterans+") OR (MH "hospitals, veterans+") OR (MH "united states department of veterans affairs+") OR (TI veteran* OR AB veteran*) OR (TI ex-service OR AB ex-service) OR (TI ex-forces OR AB ex-forces) OR (TI ex-military OR AB ex-military) OR (TI "military transitions" OR AB "military transitions") OR (TI "military to civilian" OR AB "military to civilian")) AND ((TI diet* OR AB diet*) OR (TI dietetic* OR AB dietetic*) OR (TI dietitian OR AB dietitian) OR (TI dietician OR AB dietician) OR (TI "eating pattern" OR AB "eating pattern") OR (TI "eating patterns" OR AB "eating patterns") OR (TI "eating habit" OR AB "eating habit") OR (TI "eating habits" OR AB "eating habits") OR (TI "dietary pattern" OR AB "dietary pattern") OR (TI "dietary patterns" OR AB "dietary patterns") OR (TI "dietary quality" OR AB "dietary quality") OR (TI nutrition* OR AB nutrition*) OR (TI lifestyle OR AB lifestyle) OR (TI "weight loss" OR AB "weight loss") OR (TI "weight management" OR AB "weight management") OR (TI "healthy eating" OR AB "healthy eating") OR (MH "diet+"))

Included the ‘apply equivalent subjects’ setting

Embase

('veterans'/exp OR 'veterans health'/exp OR 'veterans health service'/exp OR veteran*:ti,ab OR ‘ex service’:ti,ab OR ‘ex forces’:ti,ab OR ‘ex military’:ti,ab OR "military transitions":ti,ab OR "military to civilian":ti,ab) AND (diet*:ti,ab OR dietetic*:ti,ab OR dietitian:ti,ab OR dietician:ti,ab OR "eating pattern":ti,ab OR "eating patterns":ti,ab OR "eating habit":ti,ab OR "eating habits":ti,ab OR "dietary pattern":ti,ab OR "dietary patterns":ti,ab OR "dietary quality":ti,ab OR nutrition*:ti,ab OR lifestyle:ti,ab OR "weight loss":ti,ab OR "weight management":ti,ab OR "healthy eating":ti,ab OR 'diet'/exp OR 'diet therapy'/exp) AND [embase]/lim

Selected ‘Map to preferred term in Emtree’ and ‘Explode using narrower Emtree terms’

Cochrane CENTRAL

([mh veterans] OR [mh "veterans health"] OR [mh "veterans health services"] OR [mh "united states department of veterans affairs"] OR veteran*:ti,ab OR ex-service:ti,ab OR ex-forces:ti,ab OR ex- military:ti,ab OR "military transitions":ti,ab OR "military to civilian":ti,ab) AND (diet*:ti,ab OR dietetic*:ti,ab OR dietitian:ti,ab OR dietician:ti,ab OR "eating pattern":ti,ab OR "eating patterns":ti,ab OR "eating habit":ti,ab OR "eating habits":ti,ab OR "dietary pattern":ti,ab OR "dietary patterns":ti,ab OR "dietary quality":ti,ab OR nutrition*:ti,ab OR lifestyle:ti,ab OR "weight loss":ti,ab OR "weight management":ti,ab OR "healthy eating":ti,ab OR [mh diet] OR [mh “diet therapy”])

Included the function for ‘search word variations’

PsycInfo

(veterans.mh OR "veterans health".mh OR "veterans health services".mh OR "united states department of veterans affairs".mh OR veteran*.ti,ab OR ex-service.ti,ab OR ex-forces.ti,ab OR ex- military.ti,ab OR "military transitions".ti,ab OR "military to civilian".ti,ab) AND (diet*.ti,ab OR dietetic*.ti,ab OR dietitian.ti,ab OR dietician.ti,ab OR "eating pattern".ti,ab OR "eating patterns".ti,ab OR "eating habit".ti,ab OR "eating habits".ti,ab OR "dietary pattern".ti,ab OR "dietary patterns".ti,ab OR "dietary quality".ti,ab OR nutrition*.ti,ab OR lifestyle.ti,ab OR "weight loss".ti,ab OR "weight management".ti,ab OR "healthy eating".ti,ab OR diet.mh OR "diet therapy".mh)

Supplementary Material 3

Table S2. Main characteristics of studies that included MOVE! weight loss program as the intervention group or the control/comparison group, ordered and grouped by comorbid health condition (in addition to meeting eligibility criteria for MOVE! program).

Study, location and design	Health condition	Sample characteristics[a,b]	Inclusion criteria[b]	Intervention[c]	Control/ comparison[c]	Intervention mode	Intervention description	Intervention duration & contact	Adherence & Attendance
Allicock et al. (2010), USA<br><br>Quasi-experimental design	Overweight or obese	*n*=195 (92% male)<br>Age (y): 59 (12)	BMI ≥25	NC STRIDES adapted to veteran population= Healthy STRIDES/Healthy MOVE! (HSHM) [e]	MOVE! [e]	HSHM + MOVE!: group sessions, telephone, & newsletters; MOVE!: group sessions	HSHM received the standard MOVE! program plus 4 tailored newsletters and 2-4 motivational interviewing calls. Newsletters focused on fruit & vegetable consumption with additional messages related to weight control.	6 months.	Not reported
Damschroder et al. (2010), USA<br><br>Single group, pre-post feasibility study	Overweight or obese	*n*=14 (64% male)<br>Age (y): 53.8 (12.5)<br>Weight (kg): 107.6 (14.0)<br>BMI: 37.0 (4.2)<br>WC (inches): 46.5 (4.3)	VA patients eligible for MOVE!	MOVE! n=14	N/A	Individual, in-person and phone	Weekly lifestyle coaching sessions via phone. Participants logged diet and given pedometer. Used Stoplight Guide: categorises foods according to amount of fat and sugar per serving.	12 weeks, weekly sessions of interchanging intervention modes. Average phone call duration: 32.4 (7.6) minutes.	Attendance: 92% of planned sessions completed.
Romanova et al (2013), USA<br><br>Retrospective evaluation / observational cohort study	Overweight or obese	*n*=382 (87.7% male)<br>Age (y): 60 (11.1)<br>Weight (kg): 110 (23.6)<br>BMI <25=2.6%<br>BMI 25-29.9=16.5%<br>BMI 30-39.9=61%<br>BMI ≥40= 19.9%	Obese (BMI >30), or overweight (BMI 25−29.9) with comorbid conditions	MOVE!	Nil	In-person group sessions; Individual in-person as needed	Nutrition class led by dietician (2 hrs). Weekly multidisciplinary education sessions on nutrition, physical activity, behavioural health perspectives. Encouraged to set realistic, attainable goals for weight loss (0.5 to 1 kg/week).	8 weeks, 1hr weekly sessions.	Attended at least 3 sessions, *n*=377

Spring et al. (2013), USA<br><br>Randomised controlled trial	Overweight or obese	*n*=69 (85.5% male)<br>Age (y): 57.7 (11.9)<br>Weight (kg): 111.1 (15.6)<br>BMI: 36.3 (4.6)<br>WC (cm): 120.4 (11.7)	BMI 25.1-40; weight <181.4 kg; able to participate in moderate-intensity physical activity.	Mobile-enhanced support with MOVE! (+mobile) n=34	MOVE! (standard) n=35	Group sessions, telephone coaching, mobile app for self-monitoring.	Weight loss phase:<br>MOVE!: Sessions included nutrition, physical activity, behaviour change. 5% to 10% weight loss goal. +mobile: Personal digital assistant used as decision support tool to self-regulate energy intake and depict progress toward physical activity goals. Individualised guidance based on data provided by telephone coaching.<br><br>Maintenance phase:<br>Both groups: MOVE! support group sessions.	12 months. Weight loss phase (months 1-6): biweekly 1.5hr MOVE! sessions; +mobile: biweekly individual telephone coaching (10-15 mins). Maintenance phase (months 7-12): monthly sessions.	Post-intervention: *n*=69<br><br>Attendance: Mean number of meetings: +mobile=6.2 standard=5.9 (p=0.54).<br>Coaching calls (+mobile): received 74.4% of calls.<br>Attendance at all 4 outcome assessments= 73.9%.
Ahrendt et al. (2014), USA<br><br>Retrospective cohort study	Overweight or obese	*n*=120 (93% male)<br>Age (y): MOVE!=57 (10.1), CG=62 (11.1)<br>Weight(kg): MOVE!=121.9(25.6), CG=124.7 (27.4)<br>BMI: MOVE!=38.6 (7.2), CG=38.9 (7.3)	BMI >25; attended ≥1 videoconference MOVE! Group class in 2008-2010	MOVE! *n*=60	Matched overweight veterans who chose not to participate in MOVE! (CG) *n*=60	In-person group sessions delivered via video-conferencing	**Dietary component:** Mindful eating, label reading, portion control, trimming excess fat, ↑ fibre intake, ↑ H_2O intake, carbohydrate counting, calorie counting, grocery shopping tips. **Behaviour modification**: Diet and physical activity records, goal setting, problem solving, weekly weight graphs, cognitive restructuring, positive self-talk, stimulus control, stress relief.	12 weeks, 1 hour weekly class.	MOVE! Sessions completed (M ±SEM, *n*):<br>1-4 sessions= 2.1±0.3, *n*=10.<br>5-8 sessions= 6.8 ± 0.2, *n*=22.<br>9-12 sessions= 10.3 ± 0.2, *n*=28.

Damschroder et al. (2014), USA<br><br>Three-arm, 12-month randomized pragmatic effectiveness trial	Overweight or obese	*n*=481 (85% male)<br>BMI: 36.5 (range: 25.2–63.0)<br>Age (y): 55.0 (10.0)<br>Weight (kg) (M, 95% CI): ASPIRE-Phone (AP)=112.5 (109.1, 116.0)<br>ASPIRE-Group (AG)=112.4 (109.0, 115.8)<br>MOVE!=114.1 (110.4, 117.8)<br>WC (cm): AP=119.3 (116.9, 121.7)<br>AG=119.0 (116.7, 121.3)<br>MOVE!=121.3 (118.7, 124.0)	BMI ≥30, or BMI 25-29.9 with ≥1 obesity-related chronic health condition	Aspiring to Lifelong Health (ASPIRE) Small-Changes weight-loss program:<br>AP *n*=162<br>AG *n*=160	MOVE!<br>*n*=159	In-person group or phone	Intervention (ASPIRE; phone or in-person group): Participants develop goals with regular feedback and problem-solving approach to improve goal attainment; Iterative skill building to develop mastery, autonomy, and self-efficacy; Digital pedometer, Stop Light food guide, Self-monitoring log, Digital scale.	12 months.<br>AP*:* 28 sessions.<br>AG*:* 28 sessions.	Post-intervention: AP, *n*=120; AG, *n*=122; MOVE! *n*=119<br><br>Attendance: Sessions completed, M [95% CI]:<br>AP =15.2 [13.7, 16.6];<br>AG= 12.9 [11.4, 14.4];<br>MOVE!= 5.5 [4.6, 6.5]
Hoerster et al. (2014), USA<br><br>Retrospective evaluation study	Overweight or obese	*n* = 20,819 (88% male)<br>Age: 59.8 (10.1) y [c]<br>Weight: 247.6 (51.6) lbs [c]<br>PTSD, *n*= 4589<br>Other mental health conditions, *n*=7683<br>No mental health condition, n=8547	Attended ≥8 visits in 4-6 months (intense and sustained participation)<br>BMI ≥30, or, 25–29.9 and at ≥1 obesity-related comorbidity	MOVE!	Nil	Individual and group in-person and telephone counselling	MOVE! Programme	Not reported	Weights available at 6 months: *n*=16,551 (79.5%);<br>Weights available at 12 months: *n*=13,270 (63.7%)
Frankwich et al. (2015), USA<br><br>Randomised Controlled Trial	Obese or overweight	*n*=51 (% male: GT=62%; ST=85%)<br>Age (y): GT=48.4 (2.6), ST=54.6 (2.7)<br>BMI: GT=39.3 (1.3), ST=37.3 (1.4)<br>Weight (kg): GT=112.6 (4.9), ST=114.3 (4.6)	BMI ≥ 30.0, enrolled in MOVE!	Genetic therapy (GT). Nutrigenetic-guided diet allocation *n*=30	Standard therapy (ST)<br>Standard balanced diet, *n*=21	In-person group	Diet plans for both groups of Healthy Choice lunch and dinner for first 8 weeks. Matched to one of 4 possible diets (balanced, low-carb, low-fat, or Mediterranean). Received meal plan, lists of foods to incorporate, menu samples to assist diet adherence and obtain caloric goal.	24 weeks.<br>Weeks 1 - 8: diet intervention + weekly group counselling<br>Weeks 9-24: dietary intake log.	Post-intervention, week 8: GT *n*=26 (87%), ST *n*=20 (95%)<br><br>Post-intervention, week 24: GT *n*=18 (60%), ST *n*=14 (67%)

Skoyen et al. (2015), USA<br><br>Observational cohort study (retrospective clinical record review)	Overweight or obese	*n*=171 (78.4% male)<br>Age (y): 51.0 (11.68)<br>Weight (lbs): 261.1 (44.42)<br>BMI: 38.6 (5.34)	BMI≥30 or overweight (BMI 25-29.9)	TeleMOVE<br>*n*=171<br>First 90 days (1st cycle) only, *n*=65;<br>2nd cycle (180 days), *n*=106	Nil	Home-based TeleMOVE via telehealth monitor connected to phone line.	TeleMOVE: received telehealth monitor, digital scales, pedometer, MOVE! Handout. Daily educational modules. Topics included nutritional information, behavioural modification, goal-setting. Kept food logs, weighed themselves regularly, weight data electronically sent to coordinator.	90 (1st cycle) or 180 (2nd cycle) days, daily 5-minute modules.	Post-first cycle: *n*=106<br>Post-second cycle: *n*=71<br>Drop outs 1st cycle *n*=48<br>Did not enrol in 2nd cycle *n*=17<br>Drop outs 2nd cycle *n*=35<br>Average days of participation, 1st cycle: 54.5 (28.7); 2nd cycle: 167.3 (20.6)
Lutes et al (2017), USA<br><br>Extension of randomised controlled trial	Overweight or obese	*n*=332 (85.5% male)<br>Age (y): 55.98 (9.5)<br>BMI: 36.2 (6.0)	Participants who completed the 12-month assessment of ASPIRE RCT invited to participate in additional 12 months; BMI ≥ 30, or 25–29.9 with ≥1 obesity-related health condition	Aspiring to Lifelong Health weight-loss program:<br>AP *n*=105,<br>AG *n*=115	MOVE!<br>*n*=112	In-person group or phone	ASPIRE: sessions consisted of 1) progress checks toward patient-selected goals, 2) problem-solving issues related to barriers and challenges to making dietary and physical activity changes, and 3) setting goals for following 2 months.	12 months (months 13-24 of intervention (Damschroder et al., 2014)).<br>ASPIRE phone & group: 6 coaching sessions every other month, 20 minutes & 60 minutes respectively.	Post-intervention, 24 months.<br>AP: *n*=92<br>AG:*n*=104<br>MOVE!: *n*=90<br>Attendance, sessions completed M [95% CI]:<br>AG= 2.6 [2.2, 3.0], AP= 2.4 [2.0, 2.9], MOVE!=2.3 [1.4, 3.3]

Rutledge et al. (2017), USA<br><br>Observational study	Obesity	Total sample: n=699<br>Weight(kg): MOVE!=121.1(26.2); TeleMOVE=117.9 (26.3)<br><br>High adherence participants only i.e. (≥84/90 days): *n*=213<br>% male: MOVE!=87%, TeleMOVE=88%<br>Age (y): MOVE!=57.8 (10.3); TeleMOVE=53.8 (10.9)<br>Weight(kg): MOVE!=120.1(26.0); TeleMove=119.1(20.5)<br>BMI: MOVE!=38.4 (7.0), TeleMove=38.7 (5.4)	BMI ≥ 30	TeleMOVE<br>*n*=217<br><br>High Adherence analysis<br>*n*=72	MOVE!<br>*n*=482<br><br>High adherence analysis (8/8 classes)<br>*n*=141	TeleMOVE: Telehealth, MOVE!: in-person group sessions	TeleMOVE: Given telehealth monitor, digital scales, pedometer, MOVE! Handout. Daily 5-minute educational modules. Topics included nutritional information, behavioural modification, goal-setting. Kept food logs, weighed themselves daily, weight data electronically sent to coordinator for review and response.	TeleMOVE: 90 days, daily 5-minute modules.<br><br>MOVE!: 8 weeks, weekly 90-minute classes.	TeleMOVE: completed ≤84/90 days=66.9%; completed ≥84/90 days=33.1%; MOVE: attended ≤8 classes = 70.7%; attended 8/8 classes=29.3%
Jackson et al. (2017), USA<br><br>Retrospective cohort study	Obese or overweight with a weight related health condition, and no baseline CVD.	*n*=1,463,003 (92.14% male)<br>Age (y): 51.97 (11.71)<br>BMI: 31.89 (5.15)	≥1 outpatient visit/ year for ≥3 years, BMI ≥30, or BMI ≥25 with weight-related health condition, <70 yrs, no baseline CVD	MOVE!<br>*n*=1,293,755	Non-participants of MOVE! (eligible but didn't participate)<br>*n*=169,248	In-person group sessions, some offered remotely or in-person	MOVE! Programme	10 sessions	Attendance: Median no. sessions attended =2.<br>1-2 sessions= 54%;<br>3-7 sessions =26%;<br>≥8 sessions=20%.
Dahn et al (2011), USA<br><br>Quasi-experimental	Obesity and/or HT	*n*=862 (85.8% male)<br>Age (y): 54.3 (11.4)<br>BMI: SMS=34.19 (6.1), SGS=36.56 (6.9)<br>Weight (lbs): SGS approx. 7kg > SMS group, β=15.57 lbs, SE=3.44, p<0.001	BMI ≥25 or normal weight but hypertensive	SGS: Self-Management Support (SMS) session PLUS Supportive Group Sessions (SGS)<br>*n*=392	SMS only<br>*n*=470	Group sessions	SGS: Multidisciplinary group intervention, addressing nutrition, physical activity, behavioural modifications. Nutrition topics: Obesity-related health risks, benefits of small behaviour changes, nutrition basics; food logs; food guide pyramid, meal plans, shopping, label reading, maintaining success	10 weeks, weekly 90 minute sessions	

Moin et al (2017), USA<br><br>Prospective, pragmatic, non-randomised comparative effectiveness study	Overweight or obese with prediabetes	*n*=387 (89% male)<br>Age (y): 58 (range: 28-88)<br>Weight (kg): MOVE=106.3 (19.0), VA-DPP=109 (19.8)<br>BMI: MOVE=34.3 (4.9), VA-DPP=35.1 (5.4)	BMI ≥30, or overweight (BMI ≥25) with ≥ 1 obesity-related condition;<br>Prediabetes: (1) impaired fasting glucose / glucose tolerance documented in EHR; or (2) laboratory confirmation of prediabetes within previous 6 months	Lifestyle intervention, VA-Diabetes Prevention Program (VA-DPP), *n*=273	MOVE! *n*=114	Group in-person sessions	VA-DPP used Modified Group Lifestyle Balance curriculum. Handouts, fat and calorie-counting book, self-monitoring books for tracking food intake and physical activity, pedometer, chart for self-monitoring weight. Self-monitored weight 2x/ week, daily food intake and physical activity; Received weekly feedback re progress. Dietary focus on healthy food choices, fat intake and calories.	12 months.<br>VA-DPP: 22 sessions (16 core weekly sessions in first 6 months, then 6-monthly maintenance sessions).<br>MOVE!: 8-12 core weekly sessions, then monthly maintenance sessions.	Post-intervention: VA-DPP *n*=175, (*n*=273 in ITT analysis); MOVE! *n*=113.<br>Attendance:<br>≥ 1 session: VA-DPP=73.3%, MOVE! =57.5%, p=0.002;<br>≥4 sessions: VA-DPP=57.5%, MOVE!=42.5%, p=0.007;<br>≥8 sessions: VA-DPP=42.5%, MOVE!=31%, p=0.035
Goldberg et al. (2013), USA<br><br>Randomised trial	Overweight or obese with serious mental illness (SMI)	*n*=109 (88% male)<br>Age (y): 52.0 (9.1)<br>BMI: 34.6<br>Weight (lbs): 236	SMI defined as DSM-IV diagnosis of schizophrenia, other psychotic spectrum disorder, bipolar disorder, major depression, or severe anxiety disorder;<br>BMI ≥25	MOVE! for SMI *n*=53	UC and basic informatio n (diet & exercise)<br>*n*=56	Individual and grou p (in-person)	MOVE! adapted for individuals with SMI. Psychoeducation focusing on nutritional counselling, caloric expenditure, portion control. Weekly goal setting and regular weigh-ins.	6 months.<br>Month 1: weekly individual sessions;<br>Months 2-4: weekly 60 minute group sessions;<br>Months 5-6: biweekly group review sessions; and<br>Months 2-6: monthly individual sessions	Post-intervention: UC *n*=41, MOVE! *n*=30<br>MOVE! Attendance:<br>Individual sessions:<br>4 sessions=70%<br>2-3 sessions =15%<br><2 sessions=15%<br>Mean=3.3<br>Group sessions (months 2-6):<br>≥75% of 16 group sessions=34%;<br>50%-75% sessions =15%;<br>25%-50% sessions=15%;<br><25% sessions= 36%; mean=7.4

Young et al. (2017), USA<br><br>Mixed-methods, randomised controlled trial	Obese or overweight with serious mental illness (SMI)	*n*=276<br>(% male):<br>WEBMOVE=95.8%,<br>MOVE=92.3%,<br>UC= 97.7%<br>Age (y):<br>WEBMOVE=55.5(9.2),<br>MOVE=53.8(10.1),<br>UC=54.2 (9.9)<br>BMI:<br>WEBMOVE=33.9(4.5),<br>MOVE=35.0(5.2),<br>UC=34.4 (5.6)	Diagnosis of schizophrenia, schizoaffective disorder, bipolar disorder, MDD with psychosis, or PTSD; prescribed an antipsychotic medication;<br>BMI >30, or 25-29.9 with self-reported weight gain of at least 10 pounds in last 3 months.	Computerized weight management with pe er coaching (WebMOVE) *n*=93	In-person clinician-led weight services for SMI (MOVE SMI) *n*=95; OR Education al handout on weight loss (UC) *n*=88	WebMOVE: online & telephone;<br>MOVE SMI: in-person (individual & group)<br>UC: educational handout	WebMOVE & MOVE SMI: Nutritional topics: diet plans, good nutrition, portion control, serving sizes, water and liquid calories, food labels, healthy food choices, traffic light method, fast food, eating control techniques.<br><br>UC: education on benefits of weight loss & allowed to participate in standard MOVE!	6 months.<br>WebMOVE: 30 online modules (30 minutes) + weekly peer coaching;<br>MOVE SMI: 8 individual sessions (over 6 months), 16 group sessions (in months 2-6, 60 minutes);<br>UC: handout at baseline	Post-intervention:<br>WebMOVE *n*=71,<br>MOVE SMI *n*=78,<br>UC *n*=88<br>Attendance: No. of module/sessions completed, M (SD):<br>MOVE!=14.7 (12.2) [49% of 30 modules],<br>MOVE SMI=9.7 (6.2) [41% of 24 sessions].<br>Completed 100% of modules/ sessions:<br>WebMOVE=31%<br>MOVE SMI=0%
Desouza et al. (2012), USA<br><br>Randomised pilot study	Overweight or o bese with apathy	*n*=101 (sex NR)<br>Age (y):<br>MOVE!=55.15 (10.16),<br>Methylphen=52.5 (10.42),<br>SNC=48.1 (14.29)<br>Weight(kg):<br>MOVE!=121.33 (23.45),<br>Methylphen=113.98 (21.72),<br>SNC=106.26 (25.19)<br>BMI:<br>MOVE!=38.78 (5.60),<br>Methylphen=37.58 (5.69),<br>SNC=36.06 (5.91)	Apathy (score of >40 on apathy evaluation scale), BMI>30; on stable dose of orlistat, thiazolidinediones , atypical antipsychotics or mood stabilizers for 2 months prior.	MOVE! *n*=38	Standard Nutritiona l Counsellin g (SNC), *n*=18;<br>Methylphe nidate Group (drug + MOVE) *n*=45	MOVE! - group sessions	1) MOVE! Programme<br>SNC – One initial consult with dietitian; Follow-up dictated by physician and patient.	6 months.<br>Patient visits at 1 week, 4 weeks, 13 weeks,<br>26 weeks.	Post-intervention:<br>MOVE *n*=29,<br>SNC *n*=16,<br>Methylphen *n*=34

Shiroma et al. (2020), USA	Clinically depressed	*n*=14 (86% male)<br>Age (y): 58.2 (8.5)<br>BMI: 43.9 (7.4)	BDI-II score >12; MDD by DSM-IV-TR clinician	2-week intensive MOVE	Self-Management	IMP: in-person	IMP: 2-week residential 2) program: restricted access to food, exercise	IMP: 2weeks<br>SMP:10 weeks<br>90-minute	Not reported
Prospective, pilot study Comparative, observational	with severe obesity	Weight (lbs): 309.0 (71.0)	interview; BMI >40 or BMI >35 with associated comorbid conditions	residential program (IMP), *n*=7	Program (SMP), *n*=7	group and individual. SMP: group sessions	supervision, psychological support for behaviour adaptation. Low-calorie diet - target range of 150–400 kcal/d of energy expenditure with lower range representing a minimal caloric threshold of ~1000 kcal/wk from physical activity.<br>SMP: educational modules e.g. thoughts & beliefs, portions, exercise, food labels, mindfulness, stress, dining out, goals.	weekly education lectures.	

Abbreviations: ASPIRE – Aspiring to Lifelong Health weight-loss program; AG – ASPIRE-Group; AP – ASPIRE-Phone; BDI-II - Beck Depression Inventory-II; BMI - Body Mass Index; CG - control/comparison group; CVD - cardiovascular disease; DSM-IV - Diagnostic and Statistical Manual of Mental Disorder, fourth edition; DSM-IV-TR - Diagnostic and Statistical Manual of Mental Disorder, fourth edition, text revision; EHR- Electronic Health Record; GT - genetic therapy; HSHM – Healthy Strides/Healthy MOVE!; IMP – Intensive MOVE residential Program: lbs – Pounds; MDD - major depressive disorder; NC STRIDES – North Carolina Strategies for Improving Diet, Exercise, and Screening; PTSD - post-traumatic stress disorder; SGS - Supportive group sessions; SMI – Severe Mental Illness; SMS - Self Management Support; SNC – Standard Nutritional Counseling; ST - standard therapy; UC – Usual Care; VA – Veterans' Affairs; VA-DPP - Veterans Affairs Diabetes Prevention Program; WC – Waist Circumference; (y) – Years. [a] n = refers to total sample size; data are presented as mean (SD) unless otherwise stated; [b] BMI units = kg/m^2; [c] n = refers to number of individuals per group; [d] Presumed to be reported as M (SE) or NR/unclear in paper; [e] number of individuals per group not reported for this study.

Supplementary Material 3

Table S3. Health-related outcome measures in studies that included MOVE! weight loss program as the intervention group or the control/comparison group, ordered and grouped by comorbid health condition (in addition to meeting eligibility criteria for MOVE! program).

Study	Dietary Intervention	Health- related Outcome measures	Within group comparisons [a, b]	Between-group Comparisons [a, b]
Allicock et al., (2010)	Healthy STRIDES/Healthy MOVE! (HSHM) vs. MOVE!	Fruit and Vegetable Consumption (FVC)		**FVC:** HSHM=+1.7 servings, MOVE=+1.2 servings, adjusting for baseline weight, BMI and FVC (p<0.05)
Damschroder et al., (2010)	MOVE!	Weight, BMI, WC, QoL (SF-12), life satisfaction (SWLS), self-reported nutrition (NHIS Cancer Supplement Diet and Nutrition questionnaire)	**Baseline to 12 weeks: Mean difference (SD)**<br>**Weight (kg):** -3.8 (3.6), (p=0.002)<br>**BMI:** -1.2 (1.2), (p=0.003)<br>**WC (inches) :** -1.7 (1.5), (p<0.001)<br>**SF-12 MCS:** 1.2 (5.2), (p=0.42)<br>**SF-12 PCS:** 2.4 (6.9), (p=0.21)<br>**SWLS:** 0.6 (0.9), (p=0.04)<br>**Fruit & vegetable intake (servings/day)**: +2.2 (3.1), (p=0.03)<br>**Fibre intake (g/day):** 4.1 (11.9), (p=0.23)<br>**Attendance at all 12 coaching sessions (n = 8):**<br>**Weight (kg):** -5.4 (3.3), (p=0.003)<br>**BMI:** -1.6 (1.1), (p=0.004)	**Weight loss:** attendance at all 12 sessions significantly greater than <12 sessions (p=0.05)
Romanova et al., (2013) [c]	MOVE!	Weight	**Weight change 1 yr pre-enrolment (kg/yr):** +1.44 (0.47) (p = 0.003)<br>**Weight change 1 yr post-enrolment (kg/yr):** -2.19 (0.42) (p<0.001)<br>**Slope:** Pre-enrolment slope for weight change trajectory significantly different from post-enrolment slope (p<0.001)<br>**Weight change from 1-2 yrs post-enrolment (kg/yr):** 0.95 (0.57)(p=0.099)<br>**Weight change >2 yrs post-enrolment (kg/yr):** -0.54 (0.76) (p=0.48)	
Spring et al., (2013)	Mobile-enhanced support with MOVE! (+Mobile)	Weight, ≥ 5% weight loss		**Overall Weight loss:** +Mobile lost a mean 3.9 kg (3.1%) > MOVE! (95% CI 2.2, 5.5) at each time point<br>**Weight loss time points (kg), [m(95% CI)]**<br>+Mobile vs MOVE!:<br>**3 months:** -4.4 (2.7, 6.1) vs -0.86 (0.04, 1.8)<br>**6 months:** -4.5 kg (2.1, 6.8) vs -1.0 (0.7, 2.5)

				**9 months:** -3.9 (0.8, 6.9) vs 0.9 (1.1, 2.9)<br>**12 months:** -2.9 (0.5, 6.2) vs - 0.02 (2.1, 2.1)<br>**≥5% weight loss:** Significant group effect favouring +Mobile (OR=6.5; 95% CI 2.5, 18.6); did not vary significantly across time (p=0.13)<br>**% of participants achieving loss of ≥5% body weight**<br>+Mobile vs MOVE!<br>**3 months**: 36.7% vs 0%<br>**6 months:** 41.4% vs 10.7%<br>**9 months:** 33.3% vs 10.3%<br>**12 months:** 29.6% vs 14.8%.
Ahrendt et al., (2014)	MOVE!	Weight, BMI		**Baseline to 12 months:**<br>**Weight (kg):** MOVE!=-3.3 (7.5), CG=2.0 (4.4);<br>Mean unadjusted difference=–5.2 (2.5), (p<0.0001) Mean adjusted difference= –5.5 (2.7), (p<0.0001)<br>**BMI:** MOVE!=-0.97(2.4), CG=0.68(1.5), (p<0.0001)<br>**Weight for number of classes attended in 12-week period (1-4 classes, 5-8 classes, or 9-12 classes):** 9-12 classes lost more weight than 1-4 classes at 12 weeks (p<0.05); completion of ≥5 sessions had greater weight loss than CG at 12 months (p<0.05)
Damschroder et al., (2014)	ASPIRE-phone (AP) or ASPIRE-Group (AG) vs MOVE!	Weight, WC, BMI, FFQ (Fruit/vegetable servings, energy fat, fiber), LDL, HDL, Triglycerides, HbA1c, EuroQoL-5 utility, life satisfaction (SWLS), SBP, DBP	**Baseline to 12 months, expected mean change (95% CI):**<br>**Weight (kg):** AP=-1.4 (-2.4,-0.5) (p<0.01), AG=-2.8 (-3.8, -1.9) (p<0.01), MOVE!=-1.4 (-2.3, -0.4) (p<0.01)<br>**WC (cm):** AP=-2.5(-3.7, -1.3), AG=-3.5 (-4.7, -2.4), MOVE!=-1.8 (-2.9, -0.6) (all p<0.01)<br>**BMI:** AP=-0.5 (-0.7, -0.2), AG=-0.9 (-0.2, -0.6), MOVE!=-0.5 (-0.8, -0.1) (all p<0.01)<br>**% weight change**: AP= -1.3 (-2.1, -0.5), AG=-2.6 (-3.4, -1.8), MOVE!= -1.2 (-2.0, -0.4) (all p<0.01)<br>**F&V servings/day:** AP=0.5 (0.1, 0.8), (p<0.05); AG= 0.3 (-0.1, 0.6) ns, MOVE!=0.1 (-0.3, 0.4) ns<br>**Energy fat (%/day):** AP=-1.6 (-2.6, -0.6) (p<0.01), AG=-2.3 (-3.2, -1.3) (p<0.01), MOVE!=-0.8 (-1.8, 0.3) ns<br>**Fibre (g/day):** AP=1.4 (-1.1, 3-9) ns, AG=0.0 (-2.5, 2.4) ns, MOVE!=0.5 (-2.1, 3.0) ns<br>**LDL (mg/dl):** AP=-4.1 (-8.8, 0.5) ns, AG= -4.1 (-9.0, 0.8) ns, MOVE!=-0.3 (-5.1, 4.5) ns<br>**HDL (mg/dl):** AP=1.7 (0.3, 3.0) (p<0.05), AG=3.7 (2.3, 5.1) (p<0.01), MOVE!= 2.5 (1.1, 3.8) (p<0.01)<br>**Triglycerides (mg/dl):** AP=1.8 (-15.7, 19.3), AG=-0.9 (-19.3, 17.5) ns, MOVE!=-12.7 (-30.7, 5.3) ns	**Baseline to 12 months:**<br>**Weight loss:** AG vs AP (p=-0.04), AG vs MOVE! (p=0.04), AP vs MOVE! (p=0.91)<br>**≥5% body weight (% participants):** AP=21.7%, AG=23.8%, MOVE!=20.2% (p=0.79)<br>**WC (cm):** AG vs MOVE! (p<0.05), AG vs AP (p=0.23)<br>**BMI:** AG vs MOVE! (p<0.05), AG vs AP (p<0.05)<br>**% weight change:** AG vs MOVE! (p<0.05), AG vs AP (p<0.05)<br>**F&V servings/day:** all ns<br>**Energy fat (%/day):** AG vs MOVE! (p<0.05)<br>**Fiber (g/day):** all ns<br>**LDL (mg/dl):** all ns<br>**HDL:** AG vs AP (p<0.05)<br>**Triglycerides**: all ns<br>**HbA1c:** all ns<br>**EuroQoL-5 utility:** AP vs AG (p=0.03)<br>**SWLS:** all ns<br>**SBP (mmHg):** all ns<br>**DBP (mmHg):** all ns

			**HbA1c:** AP=0.0 (-0.2, 0.1) ns, AG=0.0 (-0.2, 0.1) ns, MOVE!= 0.0 (-0.2, 0.1) ns<br>**EuroQoL-5 utility:** AP=-0.01 (-0.04, 0.02) ns, AG=-0.03 (-0.05, 0.00) ns, MOVE!=-0.01 (-0.04, 0.02) ns<br>**SWLS:** AP=0.3 (0.1, 0.5) (p<0.01), AG=0.4 (0.2, 0.5) (p<0.01), MOVE!=0.5 (0.3, 0.7) (p<0.01)<br>**SBP (mmHg):** AP=-0.6 (-3.4, 2.3) ns, AG=1.8 (-1.0, 4.7) ns, MOVE!=1.3 (-1.6, 4.1) ns<br>**DBP (mmHg):** AP=-1.0 (-2.7, 0.8) ns, AG=0.6 (-1.1, 2.4) ns, MOVE!=0.0 (-1.8, 1.7) ns	
Hoerster et al., (2014)	MOVE!	Weight; ≥5% weight loss<br>**Analysed as:**<br>Total Cohort (TC), Those with post traumatic stress disorder (PTSD), Other mental health conditions (OMHC), No mental health condition (NMHC)		**Weight (lbs)**<br>**6 months**<br>TC=-6.8 (13.1), PTSD=-5.7 (13.0), OMHC=-6.3 (13.7), NMHC=-7.9 (12.4)<br>PTSD lost significantly less weight vs NMHC (mean (SD)=–0.72 (31), 95% CI (-1.32,–0.12) (p<0.05)<br>PTSD vs OMHC ns<br>**12 months**<br>TC=-6.7 (16.9), PTSD=-5.3 (16.0), OMHC=-6.2 (17.6), NMHC=-7.9 (16.5)<br>PTSD lost significantly less weight vs NMHC (mean (SD)= -1.01(0.46), 95% CI (-1.91,–0).11) (p<.05)<br>PTSD vs OMHC ns<br>**>5% body weight loss**<br>**6 months:** PTSD (23.7%) less likely to lose ≥5% of body weight than NMHC (28.7%) (OR=0.89, 95%CI (0.79, 0.99) (p<0.05)<br>OMHC (25.6%) lost significantly less weight than NMHC (mean (SD)=-0.51 (0.25), 95%CI (-1.01, -0.02) (p<0.05)
Frankwich et al., (2015)	Nutrigenetic-guided diet (GT diets: balanced, low-carb, low-fat, or Mediterranean) vs Standard balanced diet (ST)	≥5% weight loss		**8 weeks:**<br>**≥5% weight loss (% participants):** GT=26.9%, ST=35.0% (p=0.28)<br>**% weight lost:** GT=3.2% (0.6), ST=4.0% (0.7) (p=0.36)<br>**24 weeks:**<br>**≥5% weight loss (% participants):** GT=38.9%, ST=35.7% (p=0.77)<br>**% weight lost:** GT=4.3% (1.1), ST=4.4% (1.3) (p=0.93)
Skoyen et al., (2015)	TeleMOVE	Weight	**Weight (lbs)**<br>**Baseline to 90 days**<br>1st cycle only group=-8.62 (9.85); F(1,164)=126.26, p<0.0001; 2nd cycle group=-10.52 (10.32), F(1,101)=105.89, p<0.0001<br>**90 days-180 days**<br>2nd cycle group=-1.16 (6.32), p=0.11	Interaction between time x enrolment in 1 cycle vs. 2 cycles: F(1,163)=10.47, p=0.001. Interaction between enrolment in 1st cycle only vs. 2cycles x treatment: F(1,165)=11.93, p=0.001<br>**Weight (lbs) 180 days:**<br>2nd cycle group=-11.68 (12.53), 1st cycle only=-5.55 (8.23), F(1,165)= 11.55, p=0.001<br>**Outcome weights NR:** p=0.70
Lutes et al., (2017)	ASPIRE-Phone (AP); ASPIRE-Group (AG); MOVE!	Weight	**Weight change (kg) (95% CI):**<br>**24 months**<br>AP=–2.13 (–3.43, –0.83) (p=0.001), AG= –1.40 (–2.61, –0.18) (p=0.024), MOVE! –1.78 (–3.0,7 –0.49) (p=0.007)	**Weight loss at 24 months:**<br>AG vs AP p=0.56<br>AG vs MOVE! p=0.63<br>AP vs MOVE! P=0.92

Rutledge et al., (2017)	TeleMOVE; MOVE!	Weight	**Baseline to post-treatment:**<br>**Weight:** TeleMOVE (high adherence only) = -5.0 (4.5)kg, 4.2% mean weight loss (p<0.001; d=1.1)<br>MOVE! (high adherence only)=- 2.6(3.2)kg, 2.1% mean weight loss (p<0.001; d =0.80)	**Baseline to post-treatment:**<br>**Total cohort:**<br>**% achieving ≥5% weight loss:** TeleMOVE=26.6%, MOVE!=12.0% (p<0.01)<br>**Weight:** TeleMOVE=-4.3(4.5)kg, 3.6% mean weight loss; MOVE!=-2.2(3.2)kg, 1.8% mean weight loss (p<0.01)<br>**High adherence participants only:**<br>**Weight:** p<0.001; d=0.62<br>**≥5% weight loss**: TeleMOVE=43.1%, MOVE!=13.5% (p<0.001)
Jackson et al., (2017)	MOVE!	Weight, Incidence rate (IR) of CVD (CAD, CBD, PVD, HF)		**Baseline**<br>MOVE! participants had more diagnosed diabetes, hypertension, dyslipidaemia, depression, PTSD, psychoses than CG (all p<0.001)<br>**Weight change (%)** (MOVE! vs CG)<br>12 months=-0.9% vs +0.2%<br>36 months=-0.0.6% vs +0.6%<br>**Mean Observation time:** 59 months (range 1-95)<br>**Incidence Rate Associations**<br>MOVE! participation associated with:<br>17% lower incidence total CVD (HR=0.83, 95% CI(0.80, 0.86)<br>CAD HR=0.81, 95% CI(0.77, 0.86)<br>CBD HR=0.87, 95% CI(0.82, 0.92)<br>PVD HR=0.89, 95% CI(0.83, 0.94)<br>HF HR=0.78, 95% CI(0.74, 0.83)
Dahn et al., (2011)	SGS: Self-Management Support (SMS) session PLUS Supportive Group Sessions (SGS)	Weight (trajectory)	**Pre-enrolment:** average gain of 2kg/year (β=4.32lbs, SE=0.32, p<0.001)<br>**Post-enrolment:** average loss of ~1kg/year (β=−2.07lbs, SE=0.48, *p*<0.001)<br>Weight change slope pre-intervention significantly different from post-intervention (χ2(1)=7.85, p<0.01)	**Pre-enrolment weight change**: ns difference in slope between the two groups (β=0.41lbs, SE=0.44, p>0.05)<br>**Post-enrolment:**<br>**SMS only:** weight stabilized (β=0.44lbs, SE=0.78, p=0.58)<br>**SGS:** lost average 1.6 kg/year (β=−3.58 lbs, SE=0.78, p<0.001)<br>**Post Hoc analyses:**<br>**Weight (lbs) for SGS over time (Mean (SE)):**<br>0-3 months: -2.91 (0.47), p<0.001<br>3-6 months: −1.06 (0.38), p<0.01<br>6-12 months: −0.44 (0.49), p=0.37
Moin et al., (2017)	Veterans Affairs Diabetes Prevention Program (VA-DPP) cf MOVE!	Weight, HbA1c	**Weight (kg):**<br>6 months: VA-DPP=–4.1, p<0.001, MOVE!=–1.9, p=0.004<br>12 months: VA-DPP= –3.4, p<0.001, MOVE!=–2.0, p=0.07<br>**Change in HbAc1 at 12 months:** ns for both groups	**Weight:**<br>6 months: p<0.001<br>12 months: p=0.16<br>**Change in HbAc1 at 12 months:** VA-DPP=0.1%, MOVE!= 0% (p=0.41)

Goldberg et al., (2013)	MOVE! for Serious Mental Illness (SMI)	Weight, WC, Triglycerides, LDL, BP, Glucose, Block Fruit, Vegetable, and Dietary Fat Screeners, Diet and Exercise Confidence Survey (DECS), Impact of Weight on Quality of Life (IWQOL) Survey, SF-12.		**≥5% body weight loss**<br>Achieved by n=7 (6%) of total cohort<br>No significant main effect of month or month x treatment (nor for continuous weight analyses)<br>**Weight (lbs), Mean (SD):** MOVE!=-240.8 (40.1), CG= -228.1 (43.4); F=0.13, p=0.72<br>No significant group × time differences across full range of dietary management, physical activity, attitudinal, and functional measures.<br>**Group × time interaction:**<br>**Elevated WC [n (%)]:** MOVE!=19 (86), CG=24 (77); p=0.406<br>**Elevated triglycerides [n (%)]:** MOVE!=7 (35), CG=6 (24), p=0.502<br>**LDL [n (%)]:** MOVE!=9 (45), CG=7 (28), p=0.745<br>**Elevated BP [n (%)]:** MOVE!=9 (43), CG=11 (41); p=0.787<br>**High glucose [n (%)]:** MOVE!=9 (43), CG=11 (41);, p=0.787<br>**IWQOL:** MOVE!=75.1 (15.7) CG=78.9 (19.1), F=0.14 p=0.710<br>**SF-12 PCS:** MOVE!=45.2 (12.2), CG=43.9 (12.7), F=0.03, p=0.859<br>**SF-12 MCS:** MOVE!= 43.0 (15.9), CG=48.0 (9.4), F=2.30, p=0.133<br>**Block F&V Screener:**<br>**Not enough fruits or vegetables (past year), n(%)**: MOVE!=9 (30%), CG=15 (37%), p=0.678<br>**Low in fat (past year), n(%):** MOVE!=1 (3%), CG=4 (10%)<br>**Overeats, n(%):** MOVE!=10 (59%), CG=17 (71%), p=0.695<br>**Eats extremely large amounts of food at one time >once a week, n(%):** MOVE!=4 (15%), CG=5 (14), p=0.323<br>**Attitude:**<br>**DECS, diet (mean (SD) total score)):** MOVE!=80.9 (12.2), CG=78.4 (12.6); F=2.29, p=0.133<br>**Confidence to change eating and activity to control weight (0-10):** MOVE!=7.9 (1.8), CG=8.0 (2.1), F=0.07, p=0.791
Young et al., (2017)	Computerised weight management with peer coaches (WebMOVE), MOVE! SMI, UC	BMI, weight	**Baseline to 6 months**<br>**BMI change:**<br>WebMOVE: 34.9 (0.43) to 34.1 (0.43) (2.8kg weight loss), t=3.3, p=0.001<br>MOVE! SMI: 35.9 (0.40) to 35.9 (0.40), t=0.10, p=0.92<br>UC: 35.5 (0.38) to 35.6 (0.40), (0.3kg weight gain), t= −0.25, p=0.80	**Baseline to 6 months**<br>**BMI:** trend towards condition x visit effect, F=2.8, p=0.06<br>**BMI (obese only):** condition x visit effect, F=4.0, p=0.02<br>**≥5% body weight loss (obese only) =** 52/200 (26%)<br>Probability of losing ≥5% body weight significantly different between 3 treatment conditions (p=0.04) WebMOVE=22/59 (37%), MOVE! SMI=12/68 (18%), UC=18/73 (25%)<br>WebMOVE vs. MOVE! SMI (p=0.01). Other comparisons=ns
Desouza et al., (2012)	MOVE! vs Methylphenidate vs Standard Nutritional Counselling (SNC)	Weight, BMI	**Baseline to 6 months**<br>**Weight:**<br>MOVE!=−1.84 (7.14); 95%CI(-4.56, 0.87); p=0.25;<br>Methylphenidate=−4.61 (9.42); 95%CI(-7.90, -1.33); p=0.04<br>SNC=−0.60 (3.73); 95%CI(-2.59, 1.39); p=0.79<br>**BMI:**<br>MOVE=-0.61 (2.25); p=0.25<br>Methylphenidate=-1.49 (2.96); p=0.04<br>SNC=-0.12 (1.26); p=0.79	**Relative changes from baseline to 6 months:**<br>**Weight:** p=0.21<br>**BMI:** p=0.21

Shiroma et al., (2020)	Intensive (residential) MOVE! program (IMP)	Beck Depression Inventory (BDI-II), weight loss	**Depression severity (total cohort):** Significant change over time, F(3,36.77)=5.28; p<0.01<br>**Correlations:** Change in severity of depression correlated with total weight loss (r=−0.60; p=0.04) and daily energy expenditure at 12 days (r=−0.67; p=0.01), week 6 (r=−0.59; p=0.03), and week 10 (r=−0.71; p=0.01)	**BDI-II mean change**<br>**Baseline to 10 weeks:** IMP=30.3 to 20.4; SMP=21.4 to 15.4, F(1,46.75)=3.85, p=0.06<br>**At 12 days:** IMP=29.9 to 17.4; SMP=21.8 to 20.8, F(1,15.10)=9.37, p=0.01<br>**At week 6:** IMP=30.2 to 21.1; SMP=21.6 to 15.2, F(2,27.34)=4.26, p=0.03<br>**At week 10:** IMP=30.2 to 17.4, SMP=21.8 to 20.8, F(3,36.51)=2.37, p=0.09<br>**Weight loss at week 10:** F(3,10)=3.10, p=0.076, η2=0.48<br>**Weight (lbs), baseline to 12 days:**<br>IMP=313.5 (47.8) to 304.5 (92.7); SMP=304.6 (45.5) to 300.9 (93.3), F(1,12)=5.07, p=0.04<br>**Weight, day 12 to week 6; week 6 to week 10:** all ns

Abbreviations: ASPIRE – Aspiring to Lifelong Health Program; CAD = Coronary Artery Disease; CBD – Cerebrovascular Disease; CG - Control Group; Circ. - Circumference; CVD = Cardiovascular Disease; F&V – Fruit and Vegetables; GT – Genetic Test-based; HF – Heart Failure; HR – Hazard Ration; IR – Incidence Rate; IMP – Intensive MOVE! Program); lbs – Pounds; MH – Mental Health; MOVE! 23 - a 23-item questionnaire about general medical health, eating habits, exercise patterns, and barriers to weight management efforts; NHIS – National Health Interview Survey; NR – Not reported; ns – not significant; OR – Odds Ratio; PVD – Peripheral Vascular Disease; QoL - Quality of Life; SF-12 - 12-item Short Form Survey; SF-12 MCS - mental component subscale of SF-12; SF-12 PCS - physical component subscale of SF-12; SMI – Serious Mental Illness; SNC – Standard Nutritional Counselling; ST – Standard (balanced diet); SWLS - Satisfaction with Life Scale; UC – Usual Care; WC – Waist circumference; (y) – Years; 95% CI – 95% Confidence Interval. [a] Results reported as mean(SD) unless otherwise stated, [b] BMI units (kg/m^2) omitted, [c] Results reported as mean(SE)
